# STUDYING LGBT OLDER ADULTS

**DOI:** 10.1093/geroni/igae098.0108

**Published:** 2024-12-31

**Authors:** Linda Waite, Hui Liu

**Affiliations:** Chair; Discussant

## Abstract

The size of the lesbian, gay, bisexual and transgender (LGBT) population in the U.S. is estimated to be at 9 to 11 million people (about 3.5–4.5%) and is expected to grow in the future. LGBT populations persistently experience health disparities across multiple physical and mental, which widen over the life course. The study of the older LGBT population would benefit from a nationally-representative, longitudinal study with information on health outcomes but also on the social mechanisms at work that may exacerbate or ameliorate these disparities. The National Academy of Medicine (NAM) has recently called for more targeted research aimed at understanding the sources and consequences of these health disparities, particularly among older LGBT adults. The papers in this session focus on strategies to carry out such as study. O’Muircheartaigh and Schumm describe a novel strategy for recruiting such a sample; Lauderdale and Meyer present recent data mental and physical health in a small pilot study using this strategy; Johns et al. study differences in social characteristics and health for LGBT versus other older adults in two nationally-representative samples; and Azar et al. present international comparisons of these differences in the UK and the US. Together these papers add to our understanding of health and health disparities in older sexual minorities and how we might learn more about them.

